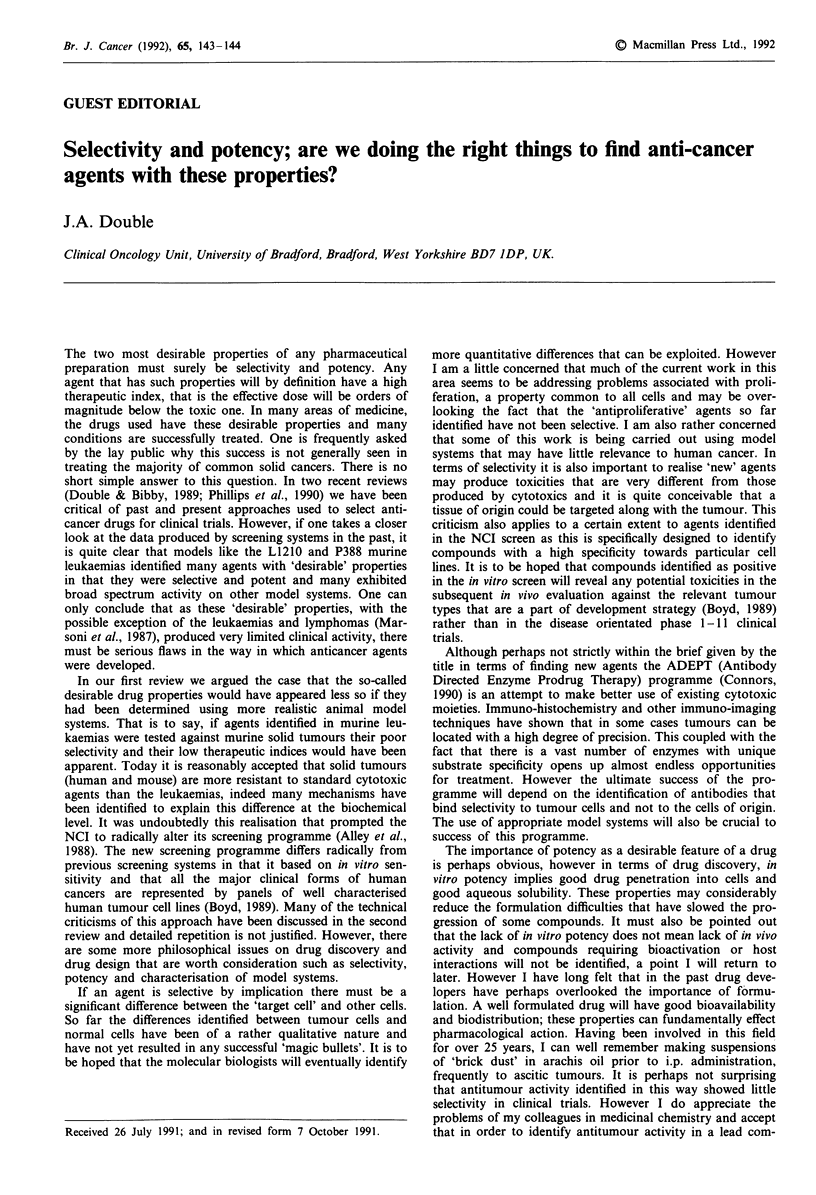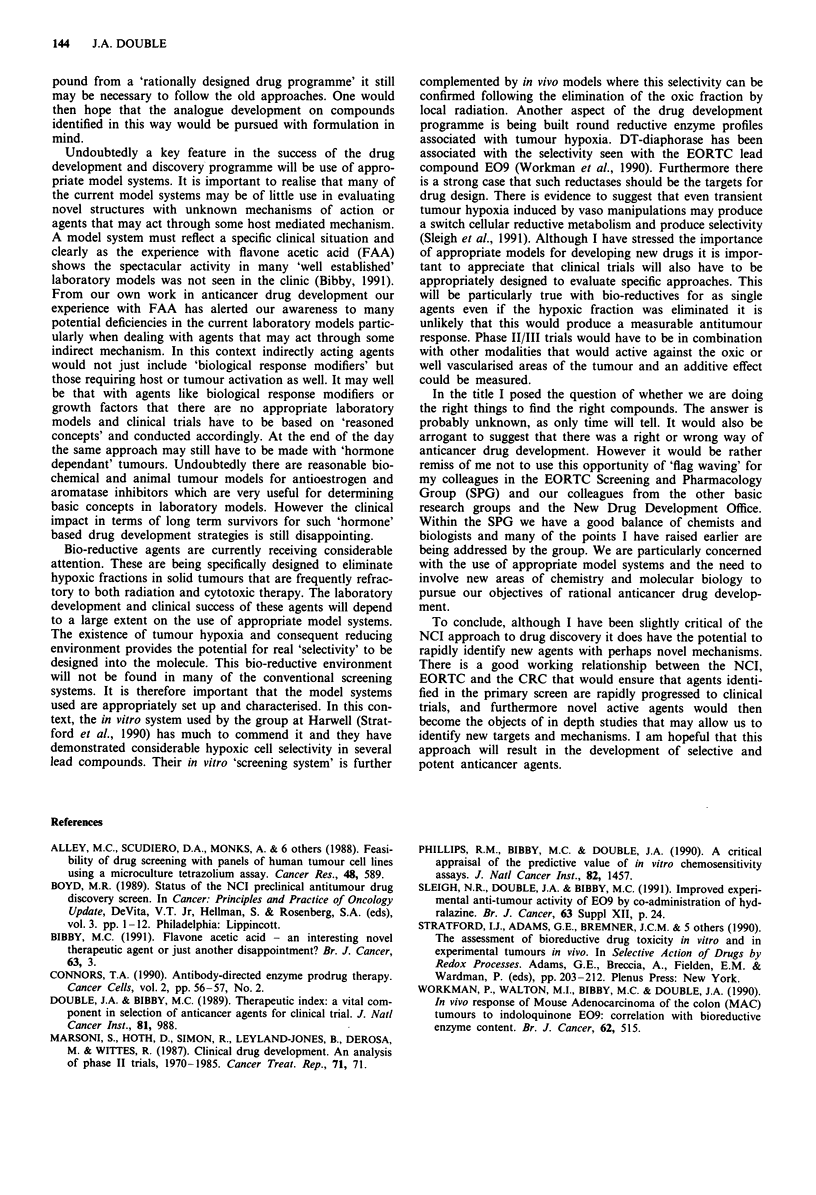# Selectivity and potency; are we doing the right things to find anti-cancer agents with these properties?

**DOI:** 10.1038/bjc.1992.30

**Published:** 1992-02

**Authors:** J. A. Double


Br. J. Cancer (1992), 65, 143–144 © Macmillan Press Ltd., 1992

GUEST EDITORIAL

### Selectivity and potency; are we doing the right things to find anti-cancer agents with these properties?

J.A. Double

*Clinical Oncology Unit, University of Bradford, Bradford, West Yorkshire BD7 1DP, UK.*

The two most desirable properties of any pharmaceutical preparation must surely be selectivity and potency. Any agent that has such properties will by definition have a high therapeutic index, that is the effective dose will be orders of magnitude below the toxic one. In many areas of medicine, the drugs used have these desirable properties and many conditions are successfully treated. One is frequently asked by the lay public why this success is not generally seen in treating the majority of common solid cancers. There is no short simple answer to this question. In two recent reviews (Double & Bibby, 1989; Phillips *et al.*, 1990) we have been critical of past and present approaches used to select anti-cancer drugs for clinical trials. However, if one takes a closer look at the data produced by screening systems in the past, it is quite clear that models like the L1210 and P388 murine leukaemias identified many agents with 'desirable' properties in that they were selective and potent and many exhibited broad spectrum activity on other model systems. One can only conclude that as these 'desirable' properties, with the possible exception of the leukaemias and lymphomas (Marsoni *et al.*, 1987), produced very limited clinical activity, there must be serious flaws in the way in which anticancer agents were developed.

In our first review we argued the case that the so-called desirable drug properties would have appeared less so if they had been determined using more realistic animal model systems. That is to say, if agents identified in murine leukaemias were tested against murine solid tumours their poor selectivity and their low therapeutic indices would have been apparent. Today it is reasonably accepted that solid tumours (human and mouse) are more resistant to standard cytotoxic agents than the leukaemias, indeed many mechanisms have been identified to explain this difference at the biochemical level. It was undoubtedly this realisation that prompted the NCI to radically alter its screening programme (Alley *et al.*, 1988). The new screening programme differs radically from previous screening systems in that it based on *in vitro* sensitivity and that all the major clinical forms of human cancers are represented by panels of well characterised human tumour cell lines (Boyd, 1989). Many of the technical criticisms of this approach have been discussed in the second review and detailed repetition is not justified. However, there are some more philosophical issues on drug discovery and drug design that are worth consideration such as selectivity, potency and characterisation of model systems.

If an agent is selective by implication there must be a significant difference between the 'target cell' and other cells. So far the differences identified between tumour cells and normal cells have been of a rather qualitative nature and have not yet resulted in any successful 'magic bullets'. It is to be hoped that the molecular biologists will eventually identify more quantitative differences that can be exploited. However I am a little concerned that much of the current work in this area seems to be addressing problems associated with proliferation, a property common to all cells and may be overlooking the fact that the 'antiproliferative' agents so far identified have not been selective. I am also rather concerned that some of this work is being carried out using model systems that may have little relevance to human cancer. In terms of selectivity it is also important to realise 'new' agents may produce toxicities that are very different from those produced by cytotoxics and it is quite conceivable that a tissue of origin could be targeted along with the tumour. This criticism also applies to a certain extent to agents identified in the NCI screen as this is specifically designed to identify compounds with a high specificity towards particular cell lines. It is to be hoped that compounds identified as positive in the *in vitro* screen will reveal any potential toxicities in the subsequent *in vivo* evaluation against the relevant tumour types that are a part of development strategy (Boyd, 1989) rather than in the disease orientated phase 1–11 clinical trials.

Although perhaps not strictly within the brief given by the title in terms of finding new agents the ADEPT (Antibody Directed Enzyme Prodrug Therapy) programme (Connors, 1990) is an attempt to make better use of existing cytotoxic moieties. Immuno-histochemistry and other immuno-imaging techniques have shown that in some cases tumours can be located with a high degree of precision. This coupled with the fact that there is a vast number of enzymes with unique substrate specificity opens up almost endless opportunities for treatment. However the ultimate success of the programme will depend on the identification of antibodies that bind selectivity to tumour cells and not to the cells of origin. The use of appropriate model systems will also be crucial to success of this programme.

The importance of potency as a desirable feature of a drug is perhaps obvious, however in terms of drug discovery, *in vitro* potency implies good drug penetration into cells and good aqueous solubility. These properties may considerably reduce the formulation difficulties that have slowed the progression of some compounds. It must also be pointed out that the lack of *in vitro* potency does not mean lack of *in vivo* activity and compounds requiring bioactivation or host interactions will not be identified, a point I will return to later. However I have long felt that in the past drug developers have perhaps overlooked the importance of formulation. A well formulated drug will have good bioavailability and biodistribution; these properties can fundamentally effect pharmacological action. Having been involved in this field for over 25 years, I can well remember making suspensions of 'brick dust' in arachis oil prior to i.p. administration, frequently to ascitic tumours. It is perhaps not surprising that antitumour activity identified in this way showed little selectivity in clinical trials. However I do appreciate the problems of my colleagues in medicinal chemistry and accept that in order to identify antitumour activity in a lead com-

Received 26 July 1991; and in revised form 7 October 1991.

pound from a 'rationally designed drug programme' it still may be necessary to follow the old approaches. One would then hope that the analogue development on compounds identified in this way would be pursued with formulation in mind.

Undoubtedly a key feature in the success of the drug development and discovery programme will be use of appropriate model systems. It is important to realise that many of the current model systems may be of little use in evaluating novel structures with unknown mechanisms of action or agents that may act through some host mediated mechanism. A model system must reflect a specific clinical situation and clearly as the experience with flavone acetic acid (FAA) shows the spectacular activity in many 'well established' laboratory models was not seen in the clinic (Bibby, 1991). From our own work in anticancer drug development our experience with FAA has alerted our awareness to many potential deficiencies in the current laboratory models particularly when dealing with agents that may act through some indirect mechanism. In this context indirectly acting agents would not just include 'biological response modifiers' but those requiring host or tumour activation as well. It may well be that with agents like biological response modifiers or growth factors that there are no appropriate laboratory models and clinical trials have to be based on 'reasoned concepts' and conducted accordingly. At the end of the day the same approach may still have to be made with 'hormone dependant' tumours. Undoubtedly there are reasonable biochemical and animal tumour models for antioestrogen and aromatase inhibitors which are very useful for determining basic concepts in laboratory models. However the clinical impact in terms of long term survivors for such 'hormone' based drug development strategies is still disappointing.

Bio-reductive agents are currently receiving considerable attention. These are being specifically designed to eliminate hypoxic fractions in solid tumours that are frequently refractory to both radiation and cytotoxic therapy. The laboratory development and clinical success of these agents will depend to a large extent on the use of appropriate model systems. The existence of tumour hypoxia and consequent reducing environment provides the potential for real 'selectivity' to be designed into the molecule. This bio-reductive environment will not be found in many of the conventional screening systems. It is therefore important that the model systems used are appropriately set up and characterised. In this context, the *in vitro* system used by the group at Harwell (Stratford *et al.*, 1990) has much to commend it and they have demonstrated considerable hypoxic cell selectivity in several lead compounds. Their *in vitro* 'screening system' is further complemented by *in vivo* models where this selectivity can be confirmed following the elimination of the oxic fraction by local radiation. Another aspect of the drug development programme is being built round reductive enzyme profiles associated with tumour hypoxia. DT-diaphorase has been associated with the selectivity seen with the EORTC lead compound EO9 (Workman *et al.*, 1990). Furthermore there is a strong case that such reductases should be the targets for drug design. There is evidence to suggest that even transient tumour hypoxia induced by vaso manipulations may produce a switch cellular reductive metabolism and produce selectivity (Sleigh *et al.*, 1991). Although I have stressed the importance of appropriate models for developing new drugs it is important to appreciate that clinical trials will also have to be appropriately designed to evaluate specific approaches. This will be particularly true with bio-reductives for as single agents even if the hypoxic fraction was eliminated it is unlikely that this would produce a measurable antitumour response. Phase II/III trials would have to be in combination with other modalities that would active against the oxic or well vascularised areas of the tumour and an additive effect could be measured.

In the title I posed the question of whether we are doing the right things to find the right compounds. The answer is probably unknown, as only time will tell. It would also be arrogant to suggest that there was a right or wrong way of anticancer drug development. However it would be rather remiss of me not to use this opportunity of 'flag waving' for my colleagues in the EORTC Screening and Pharmacology Group (SPG) and our colleagues from the other basic research groups and the New Drug Development Office. Within the SPG we have a good balance of chemists and biologists and many of the points I have raised earlier are being addressed by the group. We are particularly concerned with the use of appropriate model systems and the need to involve new areas of chemistry and molecular biology to pursue our objectives of rational anticancer drug development.

To conclude, although I have been slightly critical of the NCI approach to drug discovery it does have the potential to rapidly identify new agents with perhaps novel mechanisms. There is a good working relationship between the NCI, EORTC and the CRC that would ensure that agents identified in the primary screen are rapidly progressed to clinical trials, and furthermore novel active agents would then become the objects of in depth studies that may allow us to identify new targets and mechanisms. I am hopeful that this approach will result in the development of selective and potent anticancer agents.

### References

ALLEY, M.C., SCUDIERO, D.A., MONKS, A. & 6 others (1988). Feasibility of drug screening with panels of human tumour cell lines using a microculture tetrazolium assay. *Cancer Res.*, **48**, 589.

BOYD, M.R. (1989). Status of the NCI preclinical antitumour drug discovery screen. In *Cancer: Principles and Practice of Oncology Update*, DeVita, V.T. Jr, Hellman, S. & Rosenberg, S.A. (eds), vol. 3. pp. 1-12. Philadelphia: Lippincott.

BIBBY, M.C. (1991). Flavone acetic acid – an interesting novel therapeutic agent or just another disappointment? *Br. J. Cancer*, **63**, 3.

CONNORS, T.A. (1990). Antibody-directed enzyme prodrug therapy. *Cancer Cells*, vol. 2, pp. 56-57, No. 2.

DOUBLE, J.A. & BIBBY, M.C. (1989). Therapeutic index: a vital component in selection of anticancer agents for clinical trial. *J. Natl Cancer Inst.*, **81**, 988.

MARSONI, S., HOTH, D., SIMON, R., LEYLAND-JONES, B., DEROSA, M. & WITTES, R. (1987). Clinical drug development. An analysis of phase II trials, 1970-1985. *Cancer Treat. Rep.*, **71**, 71.

PHILLIPS, R.M., BIBBY, M.C. & DOUBLE, J.A. (1990). A critical appraisal of the predictive value of *in vitro* chemosensitivity assays. *J. Natl Cancer Inst.*, **82**, 1457.

SLEIGH, N.R., DOUBLE, J.A. & BIBBY, M.C. (1991). Improved experimental anti-tumour activity of EO9 by co-administration of hydralazine. *Br. J. Cancer*, **63** Suppl XII, p. 24.

STRATFORD, I.J., ADAMS, G.E., BREMNER, J.C.M. & 5 others (1990). The assessment of bioreductive drug toxicity *in vitro* and in experimental tumours *in vivo*. In *Selective Action of Drugs by Redox Processes*. Adams, G.E., Breccia, A., Fielden, E.M. & Wardman, P. (eds), pp. 203-212. Plenus Press: New York.

WORKMAN, P., WALTON, M.I., BIBBY, M.C. & DOUBLE, J.A. (1990). *In vivo* response of Mouse Adenocarcinoma of the colon (MAC) tumours to indoloquinone EO9: correlation with bioreductive enzyme content. *Br. J. Cancer*, **62**, 515.